# Cdc6 contributes to cisplatin-resistance by activation of ATR-Chk1 pathway in bladder cancer cells

**DOI:** 10.18632/oncotarget.9616

**Published:** 2016-05-26

**Authors:** Sansan Chen, Xinglu Chen, Gui'e Xie, Yue He, Daoyu Yan, Dianpeng Zheng, Shi Li, Xinyang Fu, Yeping Li, Xiang Pang, Zhiming Hu, Hongwei Li, Wanlong Tan, Jinlong Li

**Affiliations:** ^1^ Department of Urology, Nanfang Hospital, Southern Medical University, Guangzhou, Guangdong, China; ^2^ Institute of Biotherapy, School of Biotechnology, Southern Medical University, Guangzhou, Guangdong, China; ^3^ KingMed School of Laboratory Medicine, Guangzhou Medical University, Guangzhou, Guangdong, China

**Keywords:** Cdc6, ATR, cisplatin-resistance, bladder cancer

## Abstract

High activation of DNA damage response is implicated in cisplatin (CDDP) resistance which presents as a serious obstacle for bladder cancer treatment. Cdc6 plays an important role in the malignant progression of tumor. Here, we reported that Cdc6 expression is up-regulated in bladder cancer tissues and is positively correlated to high tumor grade. Cdc6 depletion can attenuate the malignant properties of bladder cancer cells, including DNA replication, migration and invasion. Furthermore, higher levels of chromatin-binding Cdc6 and ATR were detected in CDDP-resistant bladder cancer cells than in the parent bladder cancer cells. Intriguingly, down-regulation of Cdc6 can enhance sensitivity to CDDP both in bladder cancer cells and CDDP-resistant bladder cancer cells. Cdc6 depletion abrogates S phase arrest caused by CDDP, leading to aberrant mitosis by inactivating ATR-Chk1-Cdc25C pathway. Our results indicate that Cdc6 may be a promising target for overcoming CDDP resistance in bladder cancer.

## INTRODUCTION

Bladder cancer is the most common malignancy of urinary tract [1], which is considered a chemo-sensitive disease. The cisplatin-based combination chemotherapies, such as the GC (gemcitabine and cisplatin) and the MVAC (methotrexate, vinblastine, adriamycin and cisplatin), have greatly improved the clinical outcomes of patients with advanced or metastatic bladder cancer [2]. But the prognosis in patients with advanced or metastatic disease is still poor [3]. Development of resistance to CDDP is a great obstacle for the application of cisplatin-based therapy. Therefore, chemosensitization by reversing the chemoresistance is a promising strategy with important clinical implications for bladder cancer therapy.

The best-characterized mechanism of anti-tumor effect of CDDP is generation of DNA lesions and subsequent apoptosis [4]. DNA lesions can activate DNA damage response (DDR) which is orchestrated by multiple signal transduction processes involving cell cycle checkpoints, DNA repair, transcriptional regulation and apoptosis [5, 6]. Cell cycle checkpoint activation is essential for cell survival, as it arrests the cell cycle progress and allows time for DNA damage repair [7]. The ataxia telangiectasia and Rad3-related (ATR), a protein kinase which is recruited to the DNA lesions and activated in context of DNA damage, plays an important role in activation of cell cycle checkpoints [8–10]. ATR can phosphorylate and activate Chk1 kinase, which could inhibit Cdc25, the Cdk1-activating phosphatase, and lead to temporary cell cycle arrest [11]. If the cell cycle arrest is abolished and cell cycle progresses with the DNA lesions beyond repair, cells tend to undergo programed cell death due to genomic instability. Therefore, targeting ATR-Chk1 pathway may be of great therapeutic value for cisplatin-resistant bladder cancer [12].

Cdc6 is an essential licensing factor for DNA replication. The best-characterized function of Cdc6 is the assembly of pre-replicative complexes (pre-RC) at origins to initiate DNA replication in G1 phase [13]. Once the origins fire, Cdc6 is exported to the cytoplasm to prohibit rereplication. But a significant proportion of Cdc6 remains in the nucleus, suggesting a potentially additional function(s) other than pre-RC assembly [14]. Recently, it is reported that human Cdc6 involves in the activation of ATR signal. Cdc6 contributes to activation of cell cycle checkpoint pathway by facilitating ATR binding to chromatin. Cdc6 serves as a receptor for ATR-ATRIP complex to bind to chromatin in fission yeast [15]. In human cells, Cdc6 co-precipitates with ATR and more importantly, Cdc6 silencing impairs ATR-dependent checkpoint activation [16]. Although Cdc6 is reported to be linked to cancer development [17–20], the exactly effects of Cdc6 on malignant progression is still unknown. We examined the expression pattern of Cdc6 in bladder urothelial carcinoma and its correlation with clinicopathological factors and prognosis. We also investigated the role of Cdc6 on malignant properties in bladder cancer cell lines. In addition, we explored the correlation between Cdc6 up-regulation and ATR pathway under CDDP stress, demonstrating that Cdc6 contributes to CDDP resistance by activating ATR-Chk1-Cdc25C pathway and Cdc6 depletion can promote DNA damage and lead to aberrant mitosis, thus reverse CDDP resistance.

## RESULTS

### Characterization of Cdc6 expression in bladder cancer tissues and cell lines

**Figure 1:**
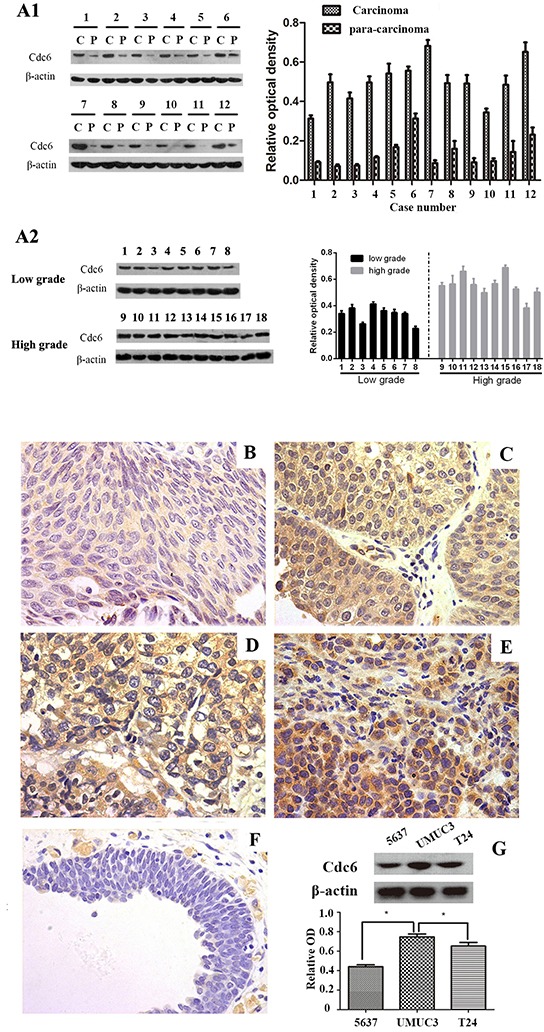
Cdc6 is highly expressed in bladder cancer tissues and cell lines **A1.** Western blot analysis of Cdc6 in tumor samples and their corresponding para-carcinoma samples from 12 patients with transitional cell bladder cancer (left). Data are expressed as optical density (OD) fold difference related to beta-actin from 3 duplicate experiments (right), c: carcinoma, p: para-carcinoma. **A2.** Western blot analysis of Cdc6 in tumor samples from low grade and high grade. **B-F.** Cdc6 expression in bladder cancer tissues and normal bladder tissues was detected by immunohistochemistry. (B) weak stain (faint yellow) in low grade, T1; (C) positive stain (yellow) in low grade, T2; (D) moderate stain (yellow) in high grade, T2; (E) strong stain (brown) in high grade, T2; (F) Negative stain in the majority of adjacent normal tissues (×400). **G.** Western blot analysis of Cdc6 expression in 5637, UMUC3 and T24 bladder cancer cell lines. Data are expressed as optical density (OD) fold difference related to beta-actin from 3 duplicate experiments, * *P*<0.05.

In order to investigate Cdc6 expression profile in bladder urothelial carcinoma, the tumor samples as well as paired adjacent bladder tissues from 12 patients with primary bladder cancer were analyzed by Western blot. Higher cdc6 expression in cancer tissues were found compared with the paired adjacent bladder tissues (Figure 1A1). We also compared Cdc6 expressions in high grade and low grade bladder cancer tissues (Figure 1A2). The results indicate that Cdc6 expression in high grade bladder cancer tissues is higher than that in low grade cancer tissues.

**Figure 2:**
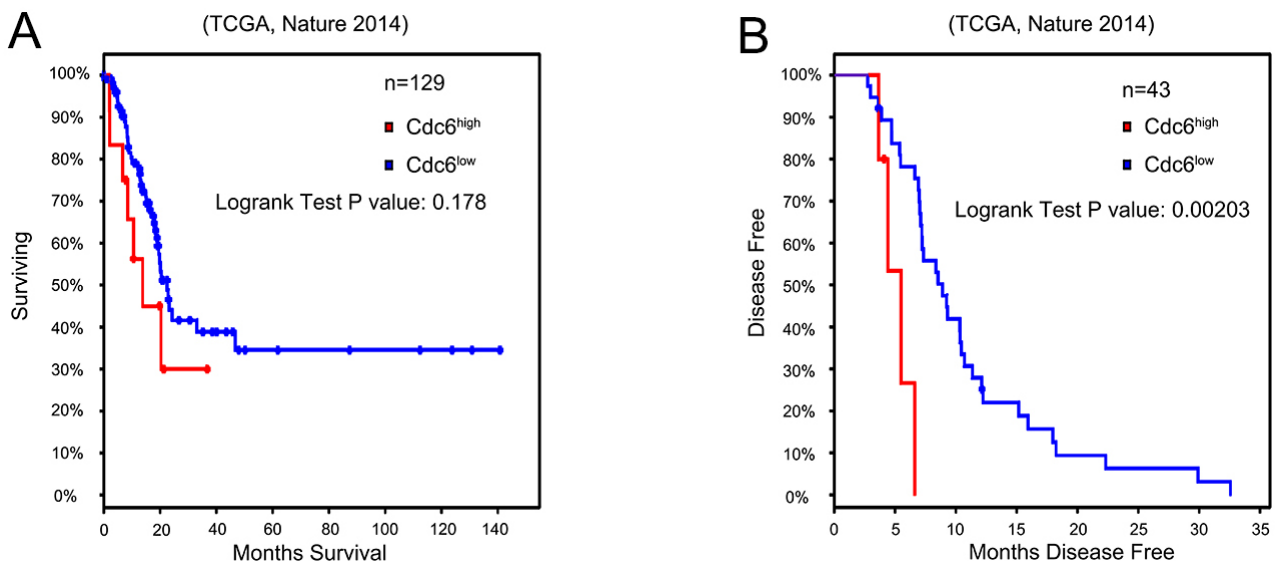
High Cdc6 expression is negatively related with prognosis of bladder urothelial cancer patients We assessed a gene expression dataset (TCGA, Nature 2014) to determine the role of Cdc6 expression in the prognosis of bladder cancer. **A.** Kaplan–Meier survival analysis of the overall survival time (n=129), P=0.178. **B.** Kaplan–Meier survival analysis of disease free survival of bladder urothelial cancer patients(n=43). Patients with higher Cdc6 expression showed significantly shorter disease free survival time than patients with lower Cdc6 expression (P=0.002).

To further investigate the relationship between Cdc6 expression and clinicopathological characteristics in bladder cancer, 115 bladder cancer samples and 50 para-carcinoma samples in tissue chips were examined by immunohistochemistry. Five representative staining results are shown in Figure 1B-1F. Cdc6 staining was localized in the cytoplasm and nucleus of tumor cells. High expression of Cdc6 was found in 81 out of 115 bladder cancer patient (70.4%), significantly higher than in normal samples (6%, 3 in 50, *P*<0.05, Table 1). As shown in Table 1, positive-cdc6 expression correlated with tumor grade (*P*=0.012). More positive-Cdc6 samples were detected in muscle invasive tumor samples (T2-T4, 75.6%) than in non-muscle-invasive tumor samples (Ta-T1, 57.6%), but with no significant statistical differences (*P*=0.055). No statistical differences of Cdc6 expression were found according to age and gender (Table 1). We next examined Cdc6 protein expression in bladder cancer cell lines UMUC3, 5637, and T24 by Western blot. As shown in Figure 1G, Cdc6 is expressed ubiquitously in bladder cancer cell lines and the strongest Cdc6 expression was detected in UMUC3 cells. Besides, we analyzed the data from bladder urothelial carcinoma study (TCGA, Nature 2014) in the cBioPortal for Cancer Genomics [21–23]. The results showed that the median survival in cases with Cdc6 up-regulation is 13.8 months, while 22.51 months in cases with no up-regulation. But the overall survival time showed no significant difference (P=0.178) (Figure 2A). The median disease free survival is significantly shorter in cases with Cdc6 mRNA up-regulation (5.49 months) than in those without Cdc6 up-regulation (8.91 months) (P=0.00203) (Figure 2B).

**Table 1:** Association of between cdc6 and various clinicopathological factors of bladder cancer patients

Characteristics		Number of patients	Cdc6 low	Cdc6 high	P value
Age group	<65	60	17	43	0.762
	>=65	55	17	38	
gender	female	25	6	19	0.491
	male	90	28	62	
Tumor stage	Non muscle invasive	33	14	19	0.055
	Muscle invasive	82	20	62	
Tumor grade	Low grade	71	27	44	0.012
	High grade	44	7	37	
Tumor vs nomal	tumor	115	34	81	0.000
	Adjacent normal tissues	50	47	3	

### Down-regulation of Cdc6 reduces DNA replication, migration and invasion in bladder cancer cell lines

**Figure 3:**
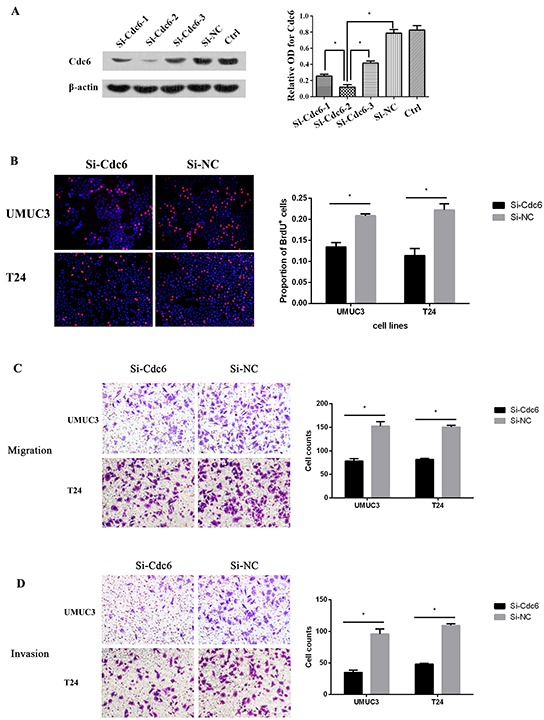
Cdc6 depletion reduced DNA replication, migration and invision in bladder cancer cells **A.** UMUC3 Cells were transfected with Cdc6 siRNA-1, 2, 3 or negative control siRNA (Si-NC) for 48 h. Cdc6 protein level was analyzed by Western blot. Beta-actin was used as the loading control. Data are expressed as optical density (OD) fold difference related to beta-actin from 3 duplicate experiments, * *P*<0.05. **B.** UMUC3 or T24 Cells were transfected with Cdc6 siRNA-2 or Si-NC for 48 h, BrdU incorporation assays were performed to evaluate DNA synthesis after transfection for 48 h; Transwell migration assay **C.** and transwell invasion assay **D.** UMUC3 or T24 Cells were transfected with Cdc6 siRNA-2 or Si-NC for 24 h, cells were plated on the upper chambers. After 24h, cells of migration and invasion were counted. Data are shown as mean ± SD of three independent experiments (right panel), * *P*<0.05.

To characterize the roles of Cdc6 on malignant properties, Cdc6-targeting siRNA or negative control siRNA (Si-NC) was transfected in UMUC3 bladder cancer cells. Among the three Cdc6-targeting siRNAs, siRNA-2 showed the most efficient inhibition (Figure 3A). So we choose siRNA-2 for the following experiments. According to the crucial role of Cdc6 in pre-replicative complexes assembly, we first examined the effect of Cdc6 depletion on DNA replication. BrdU incorporation assays revealed that the percentage of BrdU-positive cells was decreased from 22% in control group to 14% in Cdc6 RNAi group in bladder cancer UMUC3 cells (Figure 3B) (*P*<0.05). Similarly, the proportion of BrdU-positive cells was much lower in Cdc6 siRNA group than that in negative control group in T24 cells (Figure 3B).

As high Cdc6 expression correlates with high tumor grade and poor disease free survival, we reasoned that Cdc6 may have an impact on migration and invasion in bladder cancer cells. Transwell migration assays demonstrated that Cdc6 RNAi significantly decreased migration capacity in UMUC3 and T24 cells (Figure 3C). Furthermore, Cdc6 RNAi reduced the number of UMUC3 cells invading through the Matrigel by around 63% when compared to negative control (Figure 3D). Similar results were obtained in bladder cancer T24 Cells. These results indicate that Cdc6 depletion can reduce the malignant traits, including DNA replication, migration and invasion of bladder cancer cells.

### Increased chromatin-binding Cdc6 and ATR in CDDP-resistant bladder cancer cells

**Figure 4:**
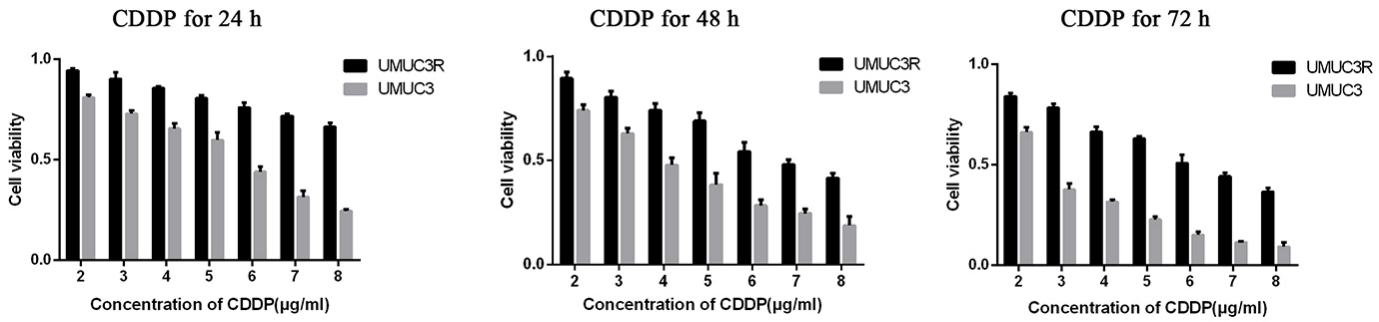
Establishment of Cisplatin-resistant UMUC3 cells (UMUC3-R) UMUC3 cells were treated intermittently with low dose of cisplatin (2μg/ml) every other day for 3 month to obtain UMUC3-R cells. UMUC3-R or UMUC3 cells were exposed to 2-8μg/ml cisplatin for 24, 48, 72 h and cell viability was quantified using Cell Titer96 Aqueous cell proliferation assay (MTS) (Promega). The UMUC3R cells treated by CDDP showed significantly higher cell viability than UMUC3 cells under the same condition. The viability ratio of UMUC3R to UMUC3 after CDDP exposure rises in a dose- and exposure time-dependent manner. The viability advantage of UMUC3-R cells indicates resistance to CDDP.

CDDP-resistant cells (UMUC3R) were obtained through desensitization by intermittent treatment of low dose of CDDP (2μg/ml) every other day for 3 months. The resistance to CDDP was confirmed by MTS assays. The UMUC3-R cells showed significantly higher viability than that of the parent UMUC3 cells after treatment with different concentrations (from 2 to 8μg/ml) of CDDP for 24, 48 or 72 hours (Figure 4). The viability ratio of UMUC3R to UMUC3 after CDDP exposure rised in a dose- and exposure time-dependent manner. This viability advantage of UMUC3R over UMUC3, especially on the condition of high CDDP concentration and long exposure time, indicates that UMUC3-R cells possess resistance to CDDP.

**Figure 5:**
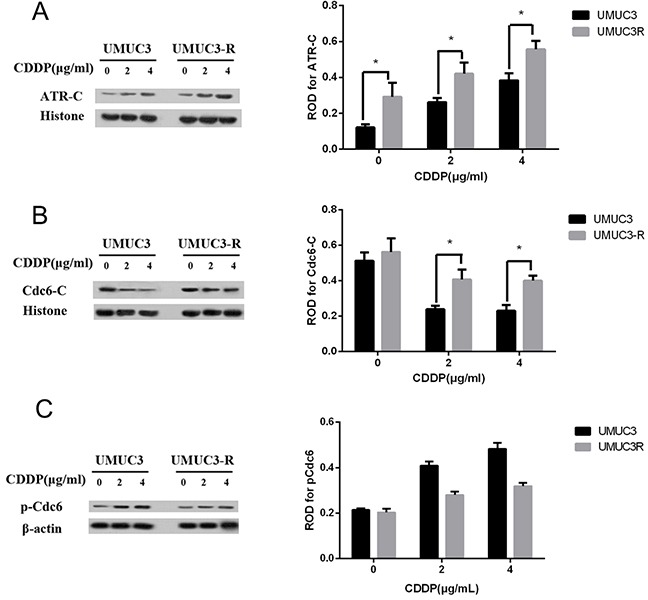
Chromatin-binding of Cdc6 and ATR are increased in UMUC3-R cells UMUC3-R or UMUC3 cells were treated with 0, 2, 4μg/ml cisplatin for 24 h. Chromatin binding ATR **A.** and Cdc6 **B.** were extracted by Chromatin-binding assays and analyzed by Western Blot. The phosphorylated Cdc6 at S74 were detected by Western blot **C.** ATR-C, Cdc6-C: chromatin-binding fraction of ATR or Cdc6. Histone H3 and beta actin were used as loading control for chromatin-binding proteins and total proteins respectively. (A2, 3) and (B2, 3) Data are presented as optical density fold difference related to loading control from three independent experiments, * *P*<0.05.

CDDP can lead to DNA damage by causing crosslinking of DNA, which ultimately triggers apoptosis [4]. Once activated by DNA damage, ATR binds to loci of DNA lesion and initiates a cascade that results in cell cycle arrest and DNA repair [10, 11]. Therefore, we hypothesize that in CDDP-resistant bladder cancer cell, more ATR bind to the damaged DNA. Chromatin binding assays were used to evaluate chromatin-binding fraction of ATR and Cdc6 in UMUC3-R cells and its parent UMUC3 cells following CDDP treatment. The results show that less ATR expressed in UMUC3 and UMUC3R cells without CDDP treatment compared to those treated by CDDP. The protein level of ATR increased following CDDP treatment in a dose dependent manner both in UMUC3-R and UMUC3 cells (Figure 5A). Notably, chromatin binding ATR in UMUC3-R was elevated greater than in non-resistant UMUC3 cells, indicating UMUC3-R cells have more powerful DNA repair capacity (Figure 5A). On the contrary, chromatin binding Cdc6 were decreased after CDDP treatment (Figure 5B). As Cdc6 mainly functions as a DNA replication initiator, we deduce that the decreased Cdc6 protein level is attributed to inhibition of DNA replication by CDDP. However, it is noteworthy that the chromatin-binding Cdc6 protein level in UMUC3R cells was remarkably higher than in UMUC3 after CDDP treatment (Figure 5B). This suggests that chromatin-binding Cdc6 may contribute to the CDDP resistance.

The phosphorylation of Cdc6 can regulate its translocation. Phosphorylation of Cdc6 at serine 74 drives translocation of Cdc6 to the cytoplasm [24]. So we analyzed phosphorylated Cdc6 in UMUC3 and UMUC3R cells after exposing to CDDP at different concentrations by Western blot. As shown in Figure 5C, the basal level of pCdc6 at S74 in UMUC3 and UMUC3R cells showed no significant differences. pCdc6 at S74 increased after CDDP treatment both in UMUC3 and UMUC3R cells, indicating CDDP treatment promotes export of Cdc6 from nucleus to cytoplasm, while the pCdc6 expression elevated greater in UMUC3 indicating that less Cdc6 were exported to cytoplasm than in UMUC3R. Accordantly, the protein level of chromatin-binding Cdc6 in UMUC3R is markedly higher compared with that in UMUC3 cells after CDDP treatment (Figure 5B).

### Down-regulation of Cdc6 enhances sensitivity of cisplatin-resistant bladder cancer cells

**Figure 6:**
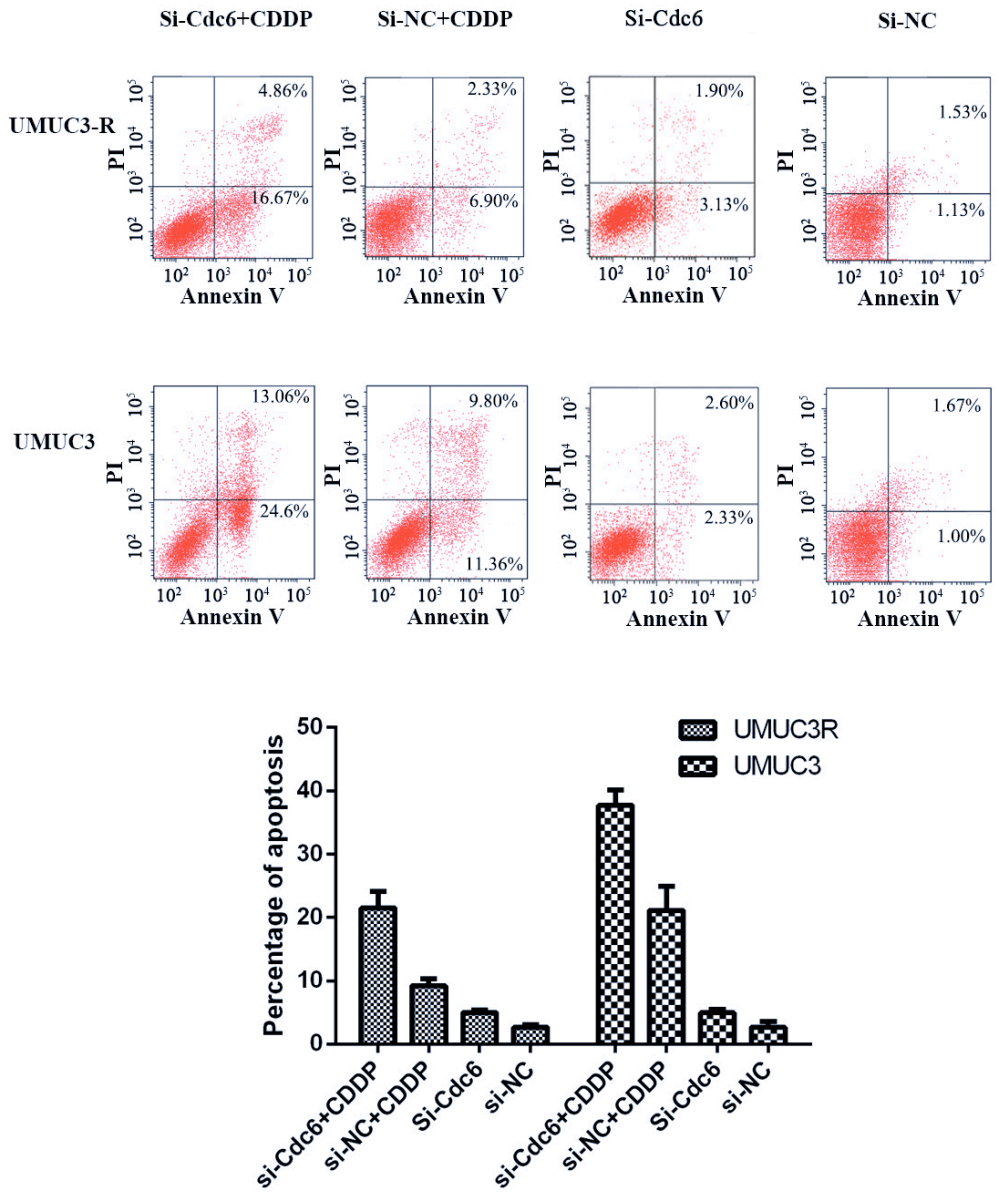
Cdc6 depletion enhances sensitivity of Cisplatin-Resistant bladder cancer cells to CDDP UMUC3-R or UMUC3 cells were treated with Cdc6 siRNA alone, 4μg/ml CDDP alone or combination for 48 hours and then stained by Annexin V and PI according to the manufacture's protocol. Cell apoptosis was examined by flow cytometry. Annexin V^+^ PI^−^ (right lower quadrant) and Annexin V^+^ PI^+^ (right upper quadrant) cells were defined as apoptotic cells. Assays were performed in triplicate.

Accumulating evidences show Cdc6 is involved in S and G2 phase cell events [25–28] and human Cdc6 can bind to ATR and is required for activation of replication checkpoint [16]. Therefore, we speculate that inhibition of Cdc6 may enhance the sensitivity of CDDP-resistant bladder cancer cells by disturbing the ATR checkpoint signal. As shown in Figure 6, CDDP (4μg/ml) induced 20% apoptosis in UMUC-3 cells versus about 9% in UMUC3-R cells, showing lower response to CDDP in UMUC3-R cells. Cdc6 depletion alone resulted in 5% and 4.9% apoptosis in UMUC3R and UMUC3 cells respectively. The combination of CDDP with Cdc6 RNAi leaded to over 21% apoptosis in UMUC3-R and 40% apoptosis in UMUC3 cells, which were significantly higher than the apoptosis rate caused by CDDP or Cdc6 siRNA alone, suggesting that Cdc6 RNAi combined with CDDP synergistically promotes apoptosis both in CDDP-resistant and non-resistant cells.

### Cdc6 depletion abolishes CDDP-induced cell cycle arrest and induces aberrant mitosis

**Figure 7:**
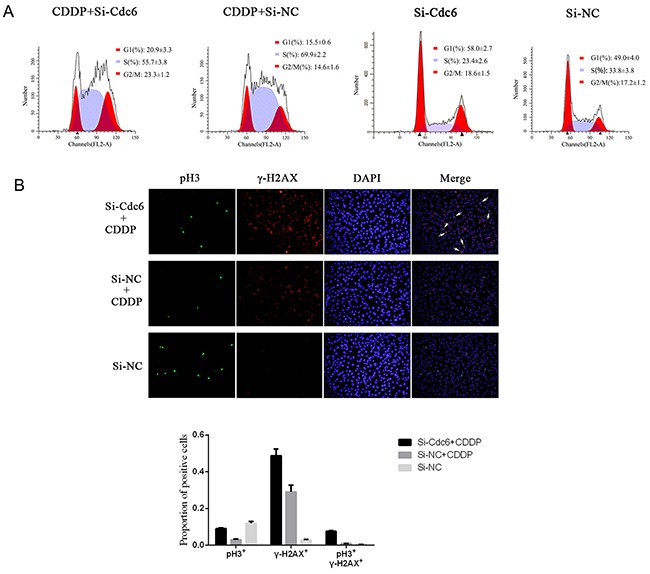
Cdc6 depletion abolishes Cisplain-induced cell cycle arrest, induces aberrant mitosis UMUC3R cells were transfected with Cdc6 RNAi (Si-Cdc6) or negative control siRNA (Si-NC), and then treated with 4μg/ml CDDP for 24h. **A.** Flow cytometry analysis of cell cycle. The distributions of cell cycle were expressed as mean±standard deviation **B.** Immunofluorescence staining was performed to detect pH3 (green) and γ-H2AX (red) in the cells, and nucleuses were visualized by DAPI staining (blue). The pH3/γ-H2AX double stained cells were marked by white arrows.

Activated ATR-dependent-checkpoint pathway can arrest cell cycle progress to repair DNA. To understand whether Cdc6 depletion could break cell cycle arrest by inhibiting ATR checkpoint pathway, we examined the cell cycle distribution of UMUC3-R cells treated by CDDP, Cdc6 RNAi or combination of both. The proportion of UMUC3R cells in G1 phase increased from 49.0% to 58.0% after Cdc6 siRNA, indicating Cdc6 depletion inhibits pre-replicative complex assembly and causes G1 arrest. What's more, CDDP resulted in S phase accumulation (70% in S, 14.6% in G2/M), and BrdU-positive cells markedly decreased after CDDP treatment (Supplementary Figure S1), indicating that CDDP inhibits DNA replication and results in S phase arrest. Interestingly, Cdc6 depletion plus CDDP caused a substantial proportion of cells abrogated S phase arrest and progressed into G2/M phase (56.7% in S, 22.3% in G2/M) (Figure 7A).

Cell cycle analysis indicated that Cdc6 depletion led to aberrant progression of cancer cells into G2/M phase under CDDP exposure. Here, to further characterize aberrant mitosis with CDDP-induced DNA damage, we assess pH3 (a mitotic marker) and γH2AX (a DNA damage marker) by immunofluorescence. The results showed few pH3- but plenty of γH2AX-stained cells in CDDP treatment group, while combination of Cdc6 RNAi with CDDP gave rise to more γH2AX-positive cells (Figure 7B), indicating that Cdc6 depletion enhances DNA damage induced by CDDP. More importantly, more pH3 positive cells were observed in the combination group than in CDDP-alone group, and the combined use of Cdc6 RNAi and CDDP led to more γ-H2AX/pH3 double stained cells, suggesting aberrant mitosis was induced (Figure 7B).

### Cdc6 depletion inhibits ATR-Chk1-Cdc25C checkpoint pathway under CDDP stress

**Figure 8:**
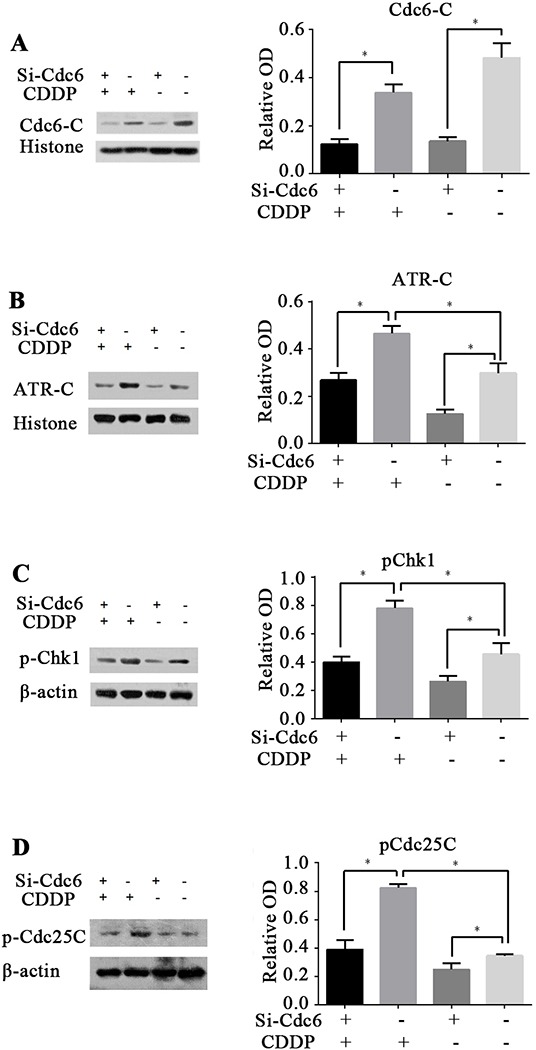
Cdc6 depletion inhibits ATR-Chk1-Cdc25 checkpoint pathway UMUC3R cells were transfected with Cdc6 RNAi (Si-Cdc6) or negative control siRNA (Si-NC), and then treated with or without CDDP (4μg/ml) for 24 h. **A, B.** Chromatin-binding of Cdc6 and ATR were analyzed by chromatin-binding assay. **C, D.** pChk1 and pCdc25C were analyzed by Western blot. Data are expressed as relative optical density (OD) fold difference related to loading control from 3 duplicate experiments, * P<0.05.

Our studies show that Cdc6 depletion abrogated S phase block induced by CDDP and resulted in aberrant mitosis with DNA damage. So we speculate that inhibition of Cdc6 may impair activation of ATR-Chk1-Cdc25C pathway and abolish cell cycle arrest, leaving DNA damage unrepaired. In accordance to previous reports, we found that CDDP can activate ATR-Chk1-Cdc25C pathway. Chromatin-binding ATR was increased after CDDP exposure, indicating ATR was loaded to the damage loci (Figure 8B). Accordingly, the p-Chk1 and p-CDC25C were also increased (Figure 8C and 8D). As expectation, after Cdc6 RNAi transfection, chromatin-binding ATR, p-Chk1 and p-CDC25C were decreased (Figure 8B,C,D), indicating that the CDDP-activated ATR-Chk1-CDC25C pathway was inhibited by Cdc6 depletion. These results indicate Cdc6 contributes to the activation of ATR-Chk1-Cdc25C pathway under DNA damage stress.

## DISCUSSION

In this study, we demonstrated elevated Cdc6 expression in bladder urothelial cancer tissues compared to normal bladder tissues. There are significant correlation between Cdc6 up-regulation and higher tumor grade (Figure 1 and Table 1). Besides, bladder cancer patients with Cdc6 up-regulation have significantly shorter disease free survival time than those with lower Cdc6 expression. These results are consistently with previous researches in other type of cancers, such as oral squamous cell carcinoma [19], lung carcinomas [18, 29], cervical carcinoma [17], gallbladder carcinoma [30], prostate cancer [20]. Considering that Cdc6 is normally absent in quiescent and differentiated cells, it could be specific markers for cancer cells. In this research, CDDP-resistant bladder cancer cells shows increased chromatin-binding ATR and Cdc6 compared to parent cells. Furthermore, Cdc6 depletion not only can inhibit DNA replication, migration and invasion, but also reverse CDDP resistance of UMUC3R and cause aberrant mitosis, probably by inactivation of ATR-Chk1-Cdc25 pathway. These results indicate that Cdc6 contributes to malignant progression of bladder cancer, and could be used as a potential anticancer target.

**Figure 9:**
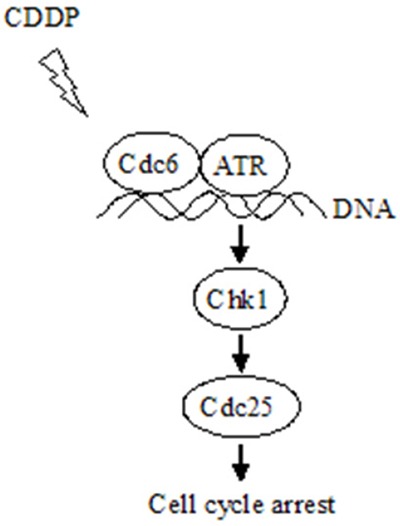
Schematic model for the mechanism that Cdc6 contributes to CDDP resistance by facilitating activation of ATR-Chk1-Cdc25C pathway Under DNA replication stress, e.g., CDDP treatment, Cdc6 collaborating with ATR binds to the damaged DNA, activates Chk1-Cdc25 pathway to arrest cell cycle allowing time for DNA repair and preventing aberrant mitosis entering without finishing DNA repair.

Dysregulation of DNA damage response (DDR) has been implicated in the CDDP-resistance in cancer treatment [31, 32]. The ATR protein kinase is a key enzyme in the DDR that maintains genomic integrity by activation of cell cycle checkpoint and DNA repair pathways [9, 33, 34]. ATR primarily acts in S and G2 phases through phosphorylation and activation of Chk1 [10], which eventually retards cell cycle progression to repair the damaged DNA. Accumulating evidence suggest that Cdc6 have additional functions other than DNA replication initiation. It has been reported that overexpression of Cdc6 can trigger checkpoint response to prevent entry into mitosis [26]. Cdc6 physically interacts with ATR, contributes to ATR activation and regulates cell cycle progression [16]. Similarly, CDC18/CDC6 is presented on the chromatin during an S-phase arrest cells, and serves as a receptor for the complex of ATR and ATR-interacting protein (ATRIP) [35]. In our study, CDDP treatment led to increase of chromatin binding ATR both in UMUC3 and UMUC3R cells (Figure 5A), suggesting the activation of ATR-dependent DNA damage response. However, CDDP treatment decreased Cdc6 protein level. This may due to the inhibition of DNA replication. Notably, CDDP-resistant UMUC3R cells expressed more chromatin-binding ATR and Cdc6 than UMUC3 after exposure to CDDP (Figure 5A and 5B), indicating Cdc6 may collaborate with ATR and contribute to CDDP resistance by facilitating activation of ATR pathway (Figure 9).

Significant progress has been made in enhancing effectiveness of chemotherapeutic agents by targeting ATR-Chk1 pathway in many cancer cells, such as colorectal cancer [36], pancreatic cancer [37], human osteosarcoma [38]. Inhibition of ATR/Chk1 pathway has been shown to sensitize cancer cells to gemcitabine, cytarabine [39] and 5-fluorouracil [40]. Here, we demonstrated that Cdc6 depletion can enhance sensitivity to CDDP by inactivating ATR-Chk1 pathway. Cdc6 downregulation increased sensitivity to CDDP both in UMUC3R and UMUC3 cells (Figure 6). Moreover, Cdc6 silence abrogated the S and S/G2 cell cycle checkpoint and caused cells to aberrantly enter mitosis with damage DNA (Figure 7). The Western blot results confirmed the inhibitory effect of Cdc6 depletion on ATR-Chk1-Cdc25C pathway (Figure 8). So inhibition of Cdc6 may be a new promising strategy to inhibit ATR pathway for killing CDDP-resistant cancer cells.

Besides the collaborating effect on ATR activation, there are several other mechanisms underlying the pro-malignance effect of Cdc6. First, Cdc6 is reported to be related with the epithelial-mesenchymal transition (EMT) and stem-like feature. A strong correlation between increased Cdc6 expression and reduced E-cadherin (a hallmark of EMT) were observed in various types of human cancers [41]. Overexpressing of Cdc6 can increase proportion of cells expressing the CD24^low^/CD44^high^ phenotype, a configuration associated with stem-like features, in A549 cells [42]. That is to say Cdc6 plays an important role in EMT; gain and maintenance of the properties of cancer stem cells, which were considered to be the key factor of tumor initiation and drug resistance. Second, Cdc6 is also reported to inhibit tumor suppressor gene. Cdc6 overexpression transcriptionally represses INK4/ARF locus [43], which encodes three important tumor suppressor genes [44]. In this paper, we showed that Cdc6 down-regulation by RNAi can attenuate the malignant properties of bladder cancer cells, including DNA replication, migration and invasion (Figure 3) and more importantly, enhance sensitivity of both UMUC3 and UMUC3R cells to CDDP (Figure 6). Therefore, Cdc6-targeting strategy to improve cancer diagnosis and treatment is a promising area, yet needing further exploration.

Cdc6 inhibition can abolish S/G2 checkpoint and induce abnormal mitosis with damage DNA indicating silencing of Cdc6 might be cytotoxic to tumors as well as to normal cells. However, the intact G1 and S/G2 cell cycle checkpoints should give survival advantage for normal cells. The ataxia telangiectasia-mutated (ATM) and ATR are the two major signal transducers in DNA damage response. Cancer cells lack G1 checkpoint because of deficiency in ATM/p53 signaling [45–50]. It has been hypothesized that cancer cells mainly rely on the ATR/Chk1 pathway to repair DNA damage [51–55]. Therefore, ATR inhibition in tumor cells can induce severe DNA damage, while normal cells with a functional G1 checkpoint are unaffected [56–59]. Therefore, targeting ATR/chk1 pathways is a useful strategy for enhancing the cancer-selective killing efficacy of DNA-damaging agents. On the ground of the multiple roles in DNA replication, migration, invasion and ATR-Chk1 checkpoint, Cdc6 may be an efficient target with unique advantage.

In conclusion, we report that Cdc6 is upregulated in bladder cancer tissues and is positively correlated with tumor grade and associated with poor disease free survival. More importantly, Cdc6 promotes CDDP resistance in bladder cancer cells by collaborating with ATR signal pathway. Accordingly, inhibition of Cdc6 could enhance cytotoxicity of CDDP in both parent and CDDP-resistant bladder cancer cells. Considering the deficiency of G1 phase checkpoint in cancer cells and the important roles of Cdc6 and ATR in chemo-resistant cancer cells, Cdc6 may be a potential specific therapeutic target for bladder cancer, especially for CDDP-resistant bladder cancer.

## MATERIALS AND METHODS

### Cell lines

Human bladder cancer UMUC3, T24 and 5637 cells were routinely maintained in MEM medium (Gibco BRL, Grand Island, NY, USA) containing 10% fetal bovine serum, penicillin (100U/ml), and streptomycin (100μg/ml) at 37°C in a balanced air humidified incubator with an atmosphere of 5% CO_2_. The CDDP-resistant subline was developed from UMUC3 by 3-months intermittent exposure to 2μg/ml CDDP every other day, and designated cell line UMUC3R.

### Patients and specimens

12 patients who underwent radical cystectomy in Nanfang hospital due to advanced bladder cancer were included in Western blot analysis. Tissue chips containing 105 patients with bladder cancer were purchased from Alenabio Co., Ltd (Xi'an, China) and shanghai Outdo Biotech co., LTD (Shanghai, China).

### Western blot

Total proteins from tissues and cells were extracted using ice-cold lysis buffer (50mM Tris-HCl pH 7.5, 150mM NaCl, 1% NP40, 1mM PMSF, and 10units/ml aprotinin) for 20min, then centrifuged at 13400 rcf for 10min at 4°C to obtain the whole cell proteins. Total proteins (about 20 μg) were separated by SDS-PAGE (6% for ATR, 10% for other proteins) and transferred onto polyvinylidene fluoride membranes and incubated overnight at 4°C with antibody against Cdc6 (Abcam), ATR, γ-H2AX (Abcam), p-Chk1 (Cell Signaling technology), pCdc25C (Cell Signaling technology), beta-actin (Cell Signaling technology) or histone H3 (Cell Signaling technology). After washing with Tris-buffered saline with Tween 20, the membranes were incubated with HRP-conjugated IgG at room temperature for 1 h. Signal detection was carried out with an ECL system (millipore, Billerica, MA, USA).

### Chromatin binding assay

Cells were harvested and resuspended in tubes with EB buffer (100mM KCl, 50mM HEPES-KOH pH 7.5, 2.5 mM MgCl_2_, 50 mM Na_4_P_2_O_7_, 0.1 mM NaVO_3_, 0.5% triton X-100) containing protease inhibitors, then set on ice for 5-10 min for incubation. The tubes were flicked to mix the solution every 2-3 min during incubation. Subsequently, 30% ice-cold sucrose containing protease inhibitors was added to the bottom of the tubes. The tubes were then spinned at 12-15 rcf, 10 min, 4°C and the supernatants were transferred to new tubes. The pellets were washed with EB buffer and flicked to dislodge the pellets from the wall of the tubes and vibrated briefly for resuspension, followed by spinning in a microfuge, 12-15 rcf, 5 min, 4°C. Combined the supernatants from the two steps (this is the non-chromosomal fraction). The pellets were resuspended with EB buffer (the pellets are the chromatin-binding fraction) and analyzed by Western blot.

### Immunohistochemistry

The expression patterns of Cdc6 in human tissue samples were analyzed using immunohistochemistry. Tissue microarray section slides were deparaffinized and heat-treated with citrate buffer, pH 6.0, for 7 min as an epitope retrieval protocol. Endogenous peroxidase activities were quenched by 3% H_2_O_2_ for 30 minutes, followed by rinsing twice in ddH_2_O and once with 0.1% Tween-20 in TBS and non-specific-binding sites were blocked with goat serum for 30 min. Sections were then incubated with the Cdc6 (Abcam) primary antibody at 4°C overnight. The sections were then thoroughly washed with 0.1% Tween-20 in TBS five times, and incubated with HRP-conjugated secondary antibody (Cell Signaling technology) for 1 hour, followed by washing five times with 0.1% Tween-20 TBS. Positive signals were visualized using 3, 3′-diaminobenzidine. Nuclei were counterstained with hematoxylin.

Two independent investigators examined all tumor slides in a blinded fashion. Immunostaining of Cdc6 was scored on a semi-quantitative scale by evaluating the intensity of the dye color and the percentage of stained cells. The staining intensity was scored as follows: 0 for negative; 1 for weak; 2 for moderate; and 3 for strong. The percentage of stained tumor cells was scored as 0-5% = 0, 5-25% = 1, 26-50% = 2, 51-75% = 3, 76-100% = 4 [60]. The two scores of each tumor sample were multiplied to give a final score of 0 to 12. Cdc6 expression was considered low or negative if the final score was less than 6; otherwise, Cdc6 expression was regarded as high or positive.

### SiRNA knockdown

Three Cdc6-targeting siRNAs (si-Cdc6-1: 5′AGGCACUUGCUACCAGCAA dTdT 3′, si-Cdc6-2: 5′CCAAGAAGGAGCACAAGAUdTdT3′, si-Cdc6-3: 5′GACAAUCAGCUGACAAUUAdTdT 3′), were purchased from Guangzhou Ribobio tech. For the transfection of siRNA, cells (5×10^5^) were seeded into 6-well plates and then were transfected with siRNA in diluted Lipofectamine containing Opti-MEM Medium (Invitrogen) according to manufacturer's protocol. Non-targeting siRNA (Si-NC) was used as the negative control.

### BrdU incorporation assays

Cells were seeded onto 22-mm diameter coverglasses placed in 6-well plates (3×10^5^ cells/coverglass). Cells were transfected with si-NC or Cdc6 siRNA for 48 h. One hour prior to fixing the cells, 10μM Brdu (Sigma chemicals) was added to the culture medium. The cells were rinsed and fixed in 4% phosphate-buffered paraformaldehyde for 10 min. Following aspiration, the cells were rinsed 3 times in PBS for 5 min and 0.2% triton X-100 was added to the specimens for 10 min. The specimens were then incubated in 4M HCl. After neutralization using PBS, the specimens were blocked in goat serum for 60 min. The blocking solution was aspirated and the specimens were incubated in diluted primary mouse-monoclonal antibody to BrdU (1:1,000, Cell Signaling Technology Inc., Beverly, MA, USA) overnight at 4°C. After rinsing 3 times in PBS for 5 min, the specimens were incubated in fluorochrome-conjugated secondary antibody diluted in PBS at room temperature in the dark and observed under fluorescent microscope. At least 1,000 cells/treatment using at least 2 coverglasses/treatment were counted, and the number of positive cells was recorded. Labeling indexes were calculated as the number of positively stained cells divided by the number of total cells.

### Transwell migration and matrigel invasion assays

Transwell systems pre-coated with Matrigel or not were used to value cell invasion and migration ability. Briefly, 5×10^4^ cells bladder cancer cells transfected with siRNA for Cdc6 or Si-NC for 24 h in serum-free medium were added to the upper chambers. The lower chambers were filled with medium that contained 10% fetal bovine serum. 24 hours later, cells invading or migrating to the outer side of the upper chamber were fixed, stained and counted. For the invasive potential of bladder cancer cells assay, inserts were pre-coated with 40 μl Matrigel (1:4 dilution; BD Biosciences, San Jose, CA). Quantification of the migration and invasion is expressed as the number of cells per high-power Field.

### Cell viability assays

To assess the response of CDDP-selected cells and non-selected cells to CDDP, the cells were plated into 96-well plates at a density of 5×10^3^ cells per well and allowed to adhere overnight in MEM. Subsequently, the cells were treated with increasing concentrations of CDDP (Sigma-Aldrich) diluted in MEM. After 48 h, cell viability was quantified using Cell Titer96 Aqueous cell proliferation assay (MTS) (Promega). The results were expressed as mean ± SD viable cells relatively to drug vehicle alone (considered as 100% viability).

### Analysis of apoptosis and cell cycle distribution by flow cytometry

Quantification of apoptosis induced by CDDP or CDDP combined with Cdc6 RNAi was performed with Annexin V and Propidium Iodide (PI) staining according to the manufacturer's (KeyGEN). Briefly, 1×10^5^ cells were resuspend in Annexin V binding buffer and stained with Annexin V-FITC and Propidium Iodide (1μg/ml). After incubation at room temperature for 15 min, the apoptotic cell was quantified by flow cytometry.

For cell cycle analysis, cells were fixed in 70% ethanol overnight at 4°C. Fixed cells were then washed once in ice-cold PBS and stained with propidium iodide (PI) staining solution (50μg/ml PI, 100μg/ml RNase, 0.05% Triton X-100 in PBS) for 30 min. PI-stained cells were then analyzed for their DNA content by using FACS (BD Biosciences, San Jose, CA, USA).

### Immunofluorescence

Cells grown on 24-well plates were fixed for 15 min in 4% (w/v) para-formaldehyde (PFA)/PBS after treatment and then permeabilized for 15 min in 0.25% (v/v) Triton X-100/PBS. After fixation and permeabilization, cells were washed three times in PBS and then blocked with goat serum for 1 h. Cells were incubated with antibodies against γ-H2AX (Abcam), pH3 (Cell signaling technology) as required for 60 min, followed by three times wash with PBS and a 60 min incubation with goat anti-mouse cy3 or goat anti-rabbit FITC secondary antibody. Fluorescence images were taken under an Olympus fluorescent microscope.

### Statistical analysis

SPSS version 13.0 for Windows was used for all statistical analyses. χ^2^ test was used to examine possible correlations between Cdc6 expression and clinicopathologic factors. Average values were expressed as mean ± standard deviation (S.D.) and statistical significance between different groups was determined by the Student's t-test. *P* values < 0.05 were considered to be significant.

## SUPPLEMENTARY FIGURE


