# Supplementary material for: Cdc6 contributes to cisplatin-resistance by activation of ATR-Chk1 pathway in bladder cancer cells
Source: Oncotarget. 2016 May 26;7(26):40362–76. doi: 10.18632/oncotarget.9616 (PMC5130013; doi:10.18632/oncotarget.9616)
Supplement: Supplementary file 1 [file oncotarget-07-40362-s001.pdf]

Cdc6 contributes to cisplatin-resistance by activation of ATR-Chk1 pathway in bladder cancer cells

SUPPLEMENTARY FIGURE

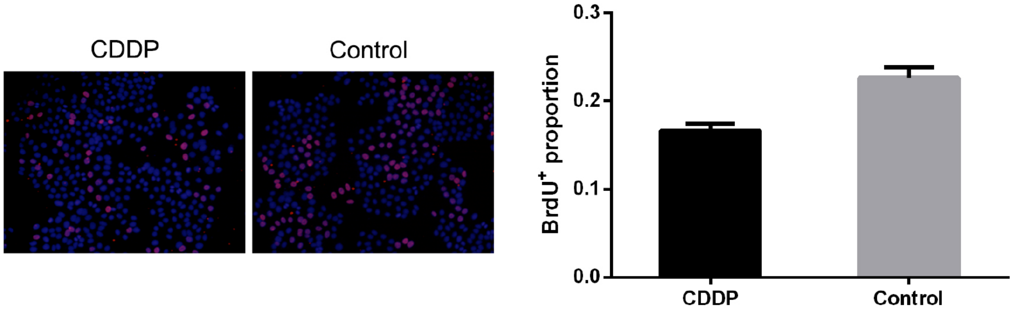

Supplementary Figure S1: CDDP inhibits DNA synthesis in UMUC3R cells. To distinguish cell arrest from cell proliferation in S phase, we examined the uptake of BrdU after CDDP treatment. UMUC3R cells were treated with 4 µg/mL CDDP for 24 h and exposed to 20µM BrdU one hour before fixation. BrdU immunodetection was administrated as described in Method section. The cells were stained by DAPI (blue) after BrdU stain. As shown in S Figure 1, CDDP treatment reduced the proportion of $BrdU^+$ (red) cells.
